# Comparison of microbial populations in the small intestine, large intestine and feces of healthy horses using terminal restriction fragment length polymorphism

**DOI:** 10.1186/1756-0500-6-91

**Published:** 2013-03-12

**Authors:** Angelika Schoster, Luis Guillermo Arroyo, Henry Rolf Staempfli, Jeffrey Scott Weese

**Affiliations:** 1 Department of Clinical Studies, Ontario Veterinary College, University of Guelph, Guelph, Canada; 2 Equine Department, Vetsuisse Faculty, Clinic for Equine Internal Medicine, University of Zurich, Winterthurerstrsse 260, Zurich, 8750, Switzerland

**Keywords:** Equine intestinal microbiota, TRFLP, Intestinal compartments, Fecal bacteria

## Abstract

**Background:**

The composition of the microbiota of the equine intestinal tract is complex. Determining whether the microbial composition of fecal samples is representative of proximal compartments of the digestive tract could greatly simplify future studies. The objectives of this study were to compare the microbial populations of the duodenum, ileum, cecum, colon and rectum (feces) within and between healthy horses, and to determine whether rectal (fecal) samples are representative of proximal segments of the gastrointestinal tract. Intestinal samples were collected from ten euthanized horses. 16S rRNA gene PCR-based TRFLP was used to investigate microbiota richness in various segments of the gastrointestinal tract, and dice similarity indices were calculated to compare the samples.

**Results:**

Within horses large variations of microbial populations along the gastrointestinal tract were seen. The microbiota in rectal samples was only partially representative of other intestinal compartments. The highest similarity was obtained when feces were compared to the cecum. Large compartmental variations were also seen when microbial populations were compared between six horses with similar dietary and housing management.

**Conclusion:**

Rectal samples were not entirely representative of intestinal compartments in the small or large intestine. This should be taken into account when designing studies using fecal sampling to assess other intestinal compartments. Similarity between horses with similar dietary and husbandry management was also limited, suggesting that parts of the intestinal microbiota were unique to each animal in this study.

## Background

The equine intestinal microbiota is complex and has an enormous impact on digestion, immune stimulation, pathogen protection and metabolism [1]. Studies investigating the equine intestinal microbiota commonly use fecal material because of the ease of sampling [2-9]. Significant differences in the microbial composition along the gastrointestinal tract have been reported in dogs and cattle [10,11], but to date few studies have assessed whether microbial populations in feces are representative of proximal compartments of the GI tract in horses. Data from other species cannot be extrapolated to horses due to significant differences in anatomy, function, and microbiota composition. None of the studies have evaluated the entire microbial population of the equine gastrointestinal tract as only specific bacterial populations or compartments were analyzed. Furthermore, equivocal conclusions were drawn [12-15]. For proper interpretation of results it is important to understand how well the bacterial populations in feces will mimic those in proximal segments of the intestine.

As the majority of microorganisms in the intestinal tract are not cultivable with standard methods [2,16], molecular methods have recently been adapted to study the composition of the intestinal microbiota [4,5,12,16]. Terminal restriction fragment length polymorphism (TRFLP) is a 16S rRNA gene PCR based method that can be used for rapid comparison of complex microbial communities, present in the equine gastrointestinal system [17]. This method can be used to compare multiple samples, to follow spatial and temporal changes in microbial populations, and to study bacterial community dynamics in the GI tract of humans [18] and animals [11,19], including horses [5].

The objectives of this study were to assess the similarities of the microbial populations of the duodenum, ileum, cecum, colon and rectum (feces) within and between healthy horses using TRFLP.

## Methods

### Description of animals

The legal and ethical requirements with regards to the humane treatment of animals described in the study were in accordance with the University of Guelph’s animal care guidelines. Between June and July 2009 intestinal contents were collected from ten horses euthanized for reasons unrelated to this study. Inclusion criteria were older than one year and euthanasia at our institution. Exclusion criteria were gastrointestinal or acute disease and current antibiotic administration. The horses originated from five farms; breeds included Standardbred, Thoroughbred, Welsh Pony and Bashkir Curly. Use and reason for euthanasia are described in Table 1. The Bashkir Curly horses originated from one farm, were group-housed on pasture 24 hours a day, and were euthanized due to equine degenerative myeloencephalopathy. The horses were allowed free access to a locally produced grass hay and water. Individual information on feed intake was not available. No dietary supplements or concentrates were fed to these six horses. Information on diet and housing of the remaining four horses was not available.

**Table 1:** Demographic data and disease history of the horses studied

**Horse**	**Farm**	**Age in years**	**Gender**	**Breed**	**Reason for euthanasia**	**Use**
1	A	3	MC	Thoroughbred	Cervical vertebral malformation	Racehorse
2	B	14	F	Welsh Pony	Pulmonary calcification	Pleasure Riding
3	C	4	F	Bashkir Curly	EDM	Breeding
4	C	3	F	Bashkir Curly	EDM	Breeding
5	D	3	M	Standardbred	Cervical vertebral malformation	Racehorse
6	C	3	F	Bashkir Curly	EDM	Breeding
7	C	2	M	Bashkir Curly	EDM	Breeding
8	C	4	F	Bashkir Curly	EDM	Breeding
9	E	3	F	Standardbred	Cervical vertebral malformation	Racehorse
10	C	4	MC	Bashkir Curly	EDM	Breeding

M = male, MC = male castrated, F = female, EDM = Equine Degenerative Myeloencephalopathy.

### Sample collection

Samples were collected from the duodenum, ileum, body of the cecum, pelvic flexure, and rectum via enterotomy within 4 h after euthanasia. Approximately 50 g of luminal intestinal content was collected with sterile forceps or a sterile spoon (depending on consistency of the ingesta). Samples were stored at −80°C until processed within 8 weeks of collection. The fecal sample was taken from the terminal part of the rectum.

### DNA extraction and PCR amplification

DNA was extracted and purified using the QIamp DNA Stool Mini Kit (Qiagen, Mississauga, ON, Canada) according to the manufacturer’s instructions. The frozen ingesta samples were thawed and thoroughly mixed by vortexing for 30 sec. One gram of ingesta was added to the initial step of the DNA extraction protocol. The DNA was stored at −20°C until use. PCR was performed within four months of DNA extraction. The hexa-chloro derivative labeled primer 5^′^GAACGCGAAGAACCTTAC3^′^ and the corresponding unlabeled primer 5^′^GGTGTGTACAAGACCC3^′^ were used for PCR analysis targeting the V6-V8 region of the 16S rRNA gene to amplify all bacteria and were adapted from a previous study in dogs [10]. PCR reactions were carried out as described in Suchodolski *et al*. [10] with minor modifications. Bovine serum albumin (BSA, Life Technologies, Burlington, ON, Canada), and tetramethylammonium chloride (tma, Life Technologies) were added to the PCR reaction. Fifty μl reactions were carried out with components as follows: 5 μl 10xPCR buffer and 5Units taq (Platinum taq polymerase®, Invitrogen, Burlington, ON, Canada), 3 μl MgCl (50 mM, Invitrogen), 2.5 μl deoxynucleoside triphosphates (dNTPs, 10 mM, GeneAmp®dNTPs, Invitrogen), 1 μl BSA (0.1 μg/μl), 5 μl tma (1 mM), and 1.5 μl of each primer (10pmol/μl). Negative PCR control samples, containing water instead of DNA, were included to rule-out contamination of PCR reagents. Purified DNA samples of *Clostridium perfringens, C. difficile, C. clostridioforme* and *Lactobacillus pentosus* were included as positive controls. These strains were obtained from our laboratory collection and were of equine origin. The PCR protocol consisted of an initial denaturation step at 94°C for 3 min, followed by 20 cycles of denaturation at 94°C for 30 sec, annealing at 54°C for 30 sec, and extension at 72°C for 1 min, and a final elongation step at 72°C for 10 min. The PCR reactions were carried out in a BioRad T100 Thermal Cycler (BioRad, Mississauga, ON, Canada). The expected size (450 bp) and purity of the PCR amplicons were assessed on 1% agarose electrophoresis gels, stained with ethidium bromide and examined under UV transillumination.

### TRFLP

PCR products were purified using Kleen Spin columns (BioRad). In a pilot experiment two restriction enzymes were assessed (*Cfo* and *Msp1,* Roche Applied Science, Laval, QC, Canada) using a subset of samples and controls. The restriction enzyme *Msp1* resulted in a larger number of T-RF peaks indicating better separation of closely related bacterial species and was chosen for the experiment. Restriction enzyme digestion was carried out according to the manufacturer’s instructions. The final volume of the digestion step consisted of 20 μl of the cleaned PCR product, 1 IU *Msp1* enzyme, 2.5 μl buffer, and 2.4 μl ddH2O. Samples were digested for 1 h at 37°C then the enzyme was inactivated by heating to 65°C for 10 min. The samples were submitted for capillary sequencing (Animal Health Laboratory, Guelph, ON, Canada). Genemapper 4.0 software was used to analyze the electropherogram. T-RF peak sizes (length in nucleotides of the DNA fragments detected) were recorded from the reported electropherogram. T-RF peaks with height less than 50 fluorescence units were considered background noise and excluded from the analysis, as is the accepted standard by the laboratory performing the TRFLP. Fragments differing by less than three base pairs were considered identical. A replicate of the PCR and TRFLP was produced.

### Statistical analysis

Between all samples, T-RF patterns were compared by calculating the Dice similarity coefficient (Cs) : Cs = (2j/[a + b]) where ‘a’ is the number of T-RF peaks unique for sample 1, ‘b’ is the number of T-RF peaks unique for sample 2, and ‘j’ is the number of common T-RF peaks [10]. Thus, two identical profiles create a Cs value of 1, whereas completely different profiles result in a Cs value of 0. Within-horse and between-horse comparison of all compartments was performed and Cs means and ranges calculated.

Samples that produced no peaks on TRFLP were removed from analysis. The variation in patterns between different intestinal compartments within and between horses was calculated by pair-wise comparison of the Cs values of all samples. The means or mean ranges of both replicates are reported unless stated otherwise.

To assess the differences between the six horses of the same breed under similar housing and dietary management and the remaining four horses the Student t-test or Mann Whitney test was performed for each compartment.

## Results

### TRFLP analysis

**Figure 1:**
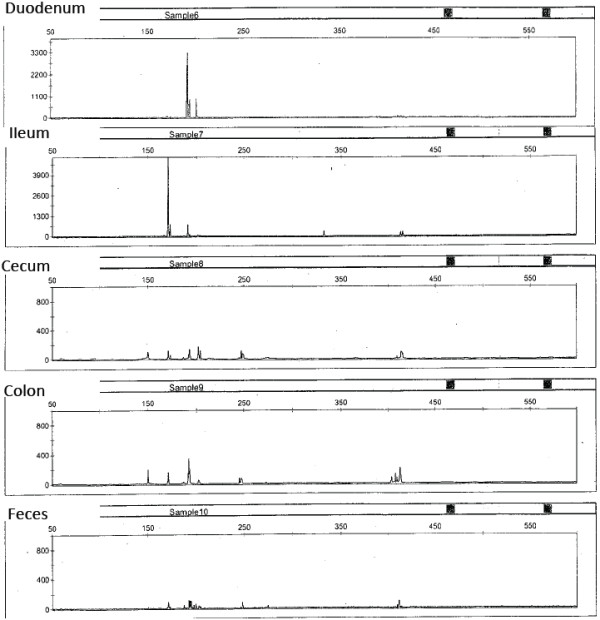
**Example of a terminal restriction fragment length polymorphism (TRFLP) electropherogram obtained from the V6-V8 16S rRNA gene PCR digested with Msp1.** The DNA was obtained from five intestinal compartments of one horse. The length of the terminal restriction fragment is plotted on the x-axis in base pairs. The intensity of the fluorescent signal of the terminal restriction fragment is plotted on the y-axis. Each peak represents a bacterial cluster present in the respective intestinal compartment. The electropherogram was accompanied by a numerical report (length of the terminal restriction fragment, height and area of the peak) from which actual numbers were drawn for analysis. Qualitative analysis for absence or presence of peaks was performed in this study to compare the richness of the equine microbiota of different gastrointestinal compartments between and within animals. The similarity coefficient (Cs) was used for comparison and was calculated as: Cs = (2j/[a + b]), where ‘a’ is the number of T-RF peaks unique for sample 1, ‘b’ is the number of T-RF peaks unique for sample 2, and ‘j’ is the number of common T-RF peaks. The scale for the y-axis depends on the maximum intensity of the fluorescent signals (height of the peak) in each sample, therefore the height of the peaks should not be compared visually. The height or area of a peak corresponds to the relative abundance of the bacterial cluster it represents and can be used for quantitative analysis.

Between 0–12 (mean: 5) T-RF peaks were generated per sample on TRFLP analysis. An example is shown in Figure 1. A total of 27 different T-RFs were detected. Five samples from four horses did not generate any T-RF peaks, were removed from analysis and are not represented in the data.

### Within-horse comparison of the gastrointestinal microbiota of 10 horses

Comparison of the T-RF peaks from the duodenum, ileum, cecum, colon and rectum within a horse demonstrated mean similarity indices from both replicates between all compartments ranging from 0.15-0.73, 0.31-0.71, 0.22-0.85, 0.13-0.88, 0.25-0.72, 0.26-0.90, 0.33-0.93, 0.20-0.74, 0–0.93, 0.27-0.89 for horses 1–10 respectively.

The mean similarity index between duodenum and ileum was 0.44, duodenum and cecum 0.39, duodenum and colon 0.35, duodenum and feces 0.39, ileum and cecum 0.42, ileum and colon 0.42, ileum and feces 0.50, cecum and colon 0.58, cecum and feces 0.67 and colon and feces 0.49. The comparison between all compartment pairs from both replicates as well as both replicates combined (mean and ranges) are summarized in Table 2.

**Table 2:** Within-horse comparison of the richness of microbiota of 5 gastrointestinal compartments of 10 horses

		**Mean**			**Ranges**					
					**Replicate 1**		**Replicate 2**		**Overall**	
		**Replicate 1**	**Replicate 2**	**Overall**	**Min**	**Max**	**Min**	**Max**	**Min**	**Max**
duodenum	ileum	0.48	0.40	0.44	0.19	0.80	0	0.75	0.10	0.78
	cecum	0.40	0.38	0.39	0	0.80	0	0.80	0	0.80
	colon	0.52	0.18	0.35	0	0.89	0	0.40	0	0.64
	feces	0.52	0.27	0.39	0	0.91	0	0.80	0	0.85
ileum	cecum	0.44	0.40	0.42	0.25	0.67	0	0.67	0.13	0.67
	colon	0.54	0.30	0.42	0.44	0.75	0	0.57	0.22	0.66
	feces	0.58	0.43	0.50	0.15	0.77	0.33	0.50	0.24	0.63
cecum	colon	0.58	0.59	0.58	0.22	0.93	0	1	0.11	0.97
	feces	0.53	0.80	0.67	0	0.83	0.67	1	0.33	0.92
colon	feces	0.56	0.42	0.49	0	0.80	0	1	0	0.90

The numbers represent the Dice similarity coefficients (Cs) between 2 compartments averaged from 10 horses. Means and ranges are reported. Replicate 1 and 2 refer to results from 2 technical replicates. Two identical profiles create a Cs value of 1, whereas completely different profiles result in a Cs value of 0.

### Comparison of rectal samples to other intestinal compartments from 10 horses

**Figure 2:**
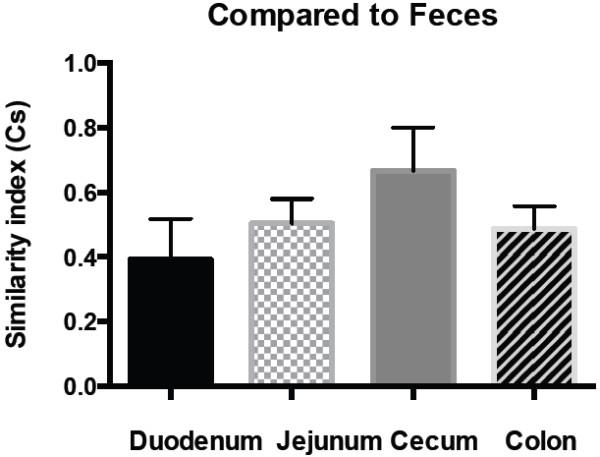
**Within-horse similarity of the gastrointestinal microbiota richness between fecal samples and other intestinal compartments based on 10 horses.** The Dice similarity coefficient (Cs) is plotted on the y-axis. The means and standard error of the mean are shown for each bar. Two identical profiles create a Cs value of 1, whereas completely different profiles result in a Cs value of 0.

Rectal samples were most similar to cecal samples. Rectal samples had a mean similarity index of 0.39, 0.50, 0.67 and 0.49 when compared to the duodenum, ileum, cecum and colon, respectively (Figure 2).

### Between-horse comparison of the gastrointestinal microbiota compared between 6 horses with similar management and 4 other horses

**Figure 3:**
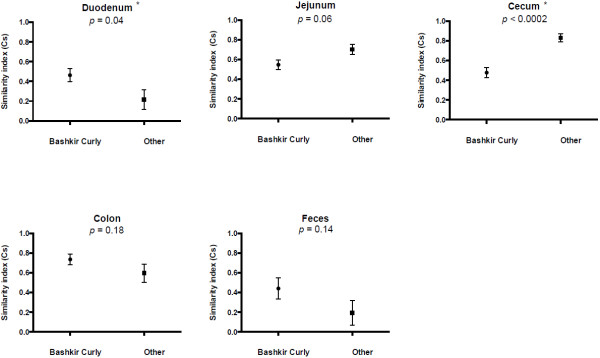
**Between-horse comparison of the intestinal microbiota of selected compartments based on 6 horses with similar dietary and housing management and 4 other horses.** The Dice similarity coefficient (Cs) is plotted on the y-axis. Two identical profiles create a Cs value of 1, whereas completely different profiles result in a Cs value of 0. The means and standard error of the mean are shown. The six Bashkir Curly horses all had similar dietary and housing management, the four other horses had unknown dietary and housing management. A significant difference in microbiota richness between similarly managed horses and other horses was seen in some compartments. * denotes significant differences (*p* < 0.05) between the six and other horses for a given compartment.

The duodenal (*p*= 0.046) and cecal (*p* = 0.002) T-RF profiles were significantly different between the six similarly managed Bashkir Curly horses compared to the remaining 4 horses. The data for the jejunum showed a trend towards significance (*p* = 0.0587). The colonic and fecal T-RF profiles did not differ significantly between the two groups with *p* values of 0.1746 and 0.1348 respectively (Figure 3).

Because of the significant differences in some compartments between the six horses under similar management and the remaining horses, calculation of differences between horses was based on the six Bashkir Curly horses only, since they originated from the same farm, were under similar housing and dietary management, and were euthanized within a period of 3 weeks. The similarity indices for the duodenum ranged between 0–0.90, ileum 0.07-0.92, cecum 0.20-0.90, colon 0.46-0.93 and feces 0.33-0.73. Compartment specific values from both replicates (means and ranges) are presented in Table 3.

**Table 3:** Between-horse comparison of the bacterial microbiota of 5 gastrointestinal compartments of 6 horses

	**Mean**			**Range**					
				**Replicate 1**		**Replicate 2**		**Overall**	
	**Replicate 1**	**Replicate 2**	**Overall**	**Min**	**Max**	**Min**	**Max**	**Min**	**Max**
Duodenum	0.41	0.46	0.44	0	0.91	0	0.89	0	0.90
Jejunum	0.64	0.42	0.53	0.13	0.83	0	1	0.07	0.92
Cecum	0.27	0.67	0.47	0	0.80	0.40	1	0.20	0.90
Colon	0.60	0.87	0.74	0.25	0.86	0.67	1	0.46	0.93
Feces	0.42	0.67	0.54	0	0.80	0.67	0.67	0.33	0.73

The horses were of the same breed, originating from the same farm with similar housing and dietary management. The numbers represent the Dice similarity coefficients (Cs) for a given compartment between the six horses. All possible horse combinations were assessed for each compartment and Cs means and ranges calculated. Mean and ranges are reported. Replicate 1 and 2 refer to results from 2 technical replicates. Two identical profiles create a Cs value of 1, whereas completely different profiles result in a Cs value of 0.

## Discussion

This study identified limited similarities in richness (Cs 0.35-0.67) of the gastrointestinal microbial populations between the small intestine, large intestine and feces within individual horses. The microbial composition in fecal samples was only partially representative of the proximal gastrointestinal compartments with the closest similarity observed between fecal and cecal samples. There were also limited similarities in richness (Cs 0.44-0.74) when comparing the same intestinal segment between horses suggesting that substantial portions of the equine intestinal microbiota are unique to each animal.

When comparing gastrointestinal compartments within a horse, the low similarity indices between compartment pairs indicated major differences in the richness of the microbiota along the gastrointestinal tract. This is consistent with results from a recent study by Dougal and co-workers (2012), who showed that that the microbial composition of the cecum is significantly different from the microbiota of the colon and feces. This is also consistent with results from other animal species; one study in cattle reported significant differences in the microbial community as ingesta travelled along the gastrointestinal tract [11]. Likewise in dogs, marked variation between compartments within dogs and within compartments between dogs was noted [10].

Rectal samples only had limited similarity to other compartments (Cs 0.39-0.67). Overall the cecum had the highest similarity index to the rectum (Cs 0.67), which could be due to separation mechanisms or actual closer similarity. These data are in agreement with findings from Milinovich *et al.* (2007), who found that relative abundance of selected fecal bacterial populations, were similar but not consistently representative of cecal bacterial populations. In contrast to the above study, the method used in our study included all bacterial species and further strengthens the suggestion that fecal samples are not consistently representative of proximal compartments.

Dougal *et al.* (2012) investigated the microbial populations of the cecum, colon and feces of three healthy horses and five healthy ponies using TRFLP. Similar to our study, the authors found that the microbial populations of the cecum were significantly different from feces; however, in contrast to our study the microbial population of the colon and the cecum were not significantly different [15]. In their study the compartment specific data was not analyzed separately for horses and ponies, and significant difference between the microbiota of horses and ponies were identified. Thus, the inclusion of ponies could have influenced the compartment specific data and could explain the difference in results of their and our study. Hastie *et al.* (2008) also found that the microbiota of the feces is representative of the distal colon; however, only certain bacterial species (*Ruminococcus flavefaciens, Fibrobacter succinogenes* and *Streptococcus bovis*) where tested, therefore potentially biasing results. Schoster *et al.* (2012) investigated the presence of *C. difficile* in various intestinal compartments of horses and found that rectal samples were only positive in 63% of animals from which *C. difficile* could be isolated from one or more proximal compartments. The data from the present study lend further credence to concerns that the fecal microbiota is not representative of the entire gastrointestinal tract. It further supports the concerns raised about the accuracy of using fecal samples for investigation and interpretation of changes in the microbiota of proximal gastrointestinal compartments, which is commonly performed [2-9].

The differences in microbiota along the gastrointestinal tract seen in our study have been observed in other studies in horses [15], as well as other animal species [10,11], and humans [20].

Along the small intestine of the horse the secretion of sodium bicarbonate and bile neutralizes the hydrochloric acid from the stomach, thus favoring acid-tolerant bacteria by raising the pH of the ingesta to approximately 7.0 by the time it reaches the large intestine. Concurrently, an increase in the total amount of cultivable bacteria is seen [21]. The ingesta entering the cecum is rich in cellulose that is only minimally digested within the small intestine. This suggests that a largely cellulolytic and proteolytic bacterial population would be present in the cecum [22]. In the colon fiber fermentation occurs, thus requiring a shift in bacterial species [23]. The right dorsal colon has limited capacity to digest fiber [24]. Soluble carbohydrate and starch bypasses digestion in the small intestine and reaches the colon [23]. Culture independent studies have reported the total bacterial population to be higher in the colon than the cecum, which could explain the higher digestibility values in these regions [25,26]. This suggests that the local microbial populations within different parts of the gastrointestinal tract are connected to the anatomy, function and composition as well as pH of the ingesta [15,27].

Significant differences in the T-RF profiles of the duodenum and cecum between the Bashkir Curly horses and the remaining horses were observed (Figure 3). Thus, similarity indices between horses were only compared based on data from the six Bashkir Curly horses, which originated from the same farm and were under similar housing and dietary management. Dietary history of the remaining four horses was not available; therefore, it cannot be speculated why these differences were present. The Bashkir Curly horses were affected by equine degenerative myeloencephalopathy, which is not known to cause changes in mentation or feeding behavior potentially altering intestinal microbial populations.

Mean similarity indices for each compartment compared between animals were low suggesting substantial differences in the microbial richness between horses in all gastrointestinal compartments. This is consistent with data from other studies that found a high level of diversity between horses [4,5,15]. Gronvold *et al.* (2010) investigated the fecal microbiota of 12 healthy horses using denaturing gradient gel electrophoresis (DGGE) and also assessed the influence of penicillin and anesthesia on the fecal microbiota. The authors demonstrated a very unique composition of the fecal microbiota for individual horses, with most of the variation attributable to the statistical effect of the individual horse, not anesthesia or penicillin administration. Willing *et al.* (2009) reported that consistency of the microbial composition of feces within an individual fed different diets was on average 73%, with a significant effect of horse on the diversity and stability of the gastrointestinal microbiota. Taken in relation to the results of these previous studies, our results support that the equine microbiota has a substantial portion unique to each animal. Such variability needs to be taken into account when comparing treatment effects between horses.

TRFLP allows for rapid assessment of complex bacterial communities, such as those present in the equine gastrointestinal system, and permit rapid comparison of the community richness; however, there are limitations. TRFLP cannot estimate bacterial community evenness or abundance, parameters necessary to evaluate bacterial diversity [17]. Phylogeny techniques based on 16S rRNA can identify strains to a family or genus level, but are of limited utility for differentiation of species as organisms can have almost identical 16S rRNA gene sequences and still belong to separate species based on DNA hybridization [28]. A additional limitation specific to the TRFLP technique is the inability to link a peak on the electropherogram to a specific bacterial family or genus without supplementary tests which could be accomplished in tandem with 16S rDNA clone library analysis [19]. Bacterial species identification is generally limited by the lack of a generally accepted bacterial species concept. Despite the existence of a bacterial species definition, which governs when a strain can be called a new species based on DNA hybridization or 16S rRNA gene sequencing, a true concept of what constitutes a bacterial species taking into account horizontal gene transfer, phenotyping and genotypic characteristics has not been agreed upon. Recently, a genomic phylogenetic species concept for the taxonomy of prokaryotes has been proposed. This concept is based on integrating information obtained from new molecular techniques such as multilocus sequence typing and genomic approaches into the existing phylogenetic concept [28].

These recent advances in the field of metagenomics make this a more attractive option for future studies to conclusively determine the differing composition of the equine microbiota in different intestinal compartments. This technique has recently been used to characterize the fecal microbiota of two healthy horses, which showed that the equine microbiota is more diverse than the human microbiota, but less diverse than the gastrointestinal microbiota of cattle [2]. Metagenomic analysis was also recently used by Costa *et al.* (2012) assessing the microbiota of healthy horses and horses affected with acute colitis. Their study showed profound differences in the microbiome of diseased horses compared to healthy horses. Determining the specific composition of the gastrointestinal microbiota was not an objective of this study; therefore, TRFLP was deemed adequate.

Comparable studies in other animals and humans have reported an average of 46–50 peaks per sample [11,18]. Similar results were expected in horses, as a prior equine study had shown 138 different TR-F peaks, although the number per sample was not reported [5]. Therefore, the low number of peaks in our study was surprising. Previous studies considered every peak as a separate one, as opposed to clustering T-RF peaks based on controls and thus obtained a larger number of T-RF peaks. This could have partially accounted for the low number of peaks seen in this study [11]. Based on results from controls we chose to group peaks within three base pairs. However, others that used similar clustering of T-RF peaks still obtained approximately 80 unique T-RF peaks from fecal samples, indicating that this alone does not account for the low number of peaks seen in our study [18]. The low number of peaks could indicate insufficient separation of very similar bacterial species, suggesting that the primer and restriction enzyme combination used lacked discriminatory power, even though two different enzymes were evaluated in a pilot trial and the enzyme creating larger numbers of peaks was chosen. Five samples did not create any T-RF peaks despite of a positive PCR result, indicating that the DNA was lost during restriction enzyme digestion or TRFLP analysis.

The primers for the amplification of the 16S rRNA gene for all bacteria were adapted from a previous study in dogs [10]. The microbiota of dogs is likely significantly different from the equine microbiota, given the differences in diet, digestion and fermentation; however, there are few studies published on the overall composition of the equine microbiota [2,8], therefore primers could not be chosen according to this knowledge. The 16S rRNA gene is known to be extremely conserved among all bacterial species known to date, therefore it was chosen as target for this study [28].

The sample size of 10 horses in our study was comparable to similar previous studies in horses [13,15], and despite sample size limitations, this study contributes with new findings regarding the equine gastrointestinal microbiota.

There was a large variation between the two technical replicates in some samples. This could potentially reflect the inherent bias of the methodology (biased PCR amplification) or insufficient restriction enzyme digestion; however, some variations between technical replicates can be expected.

## Conclusion

In conclusion this study showed that the microbial populations differ between gastrointestinal compartments of healthy horses. Fecal samples showed only moderate similarity to other gastrointestinal compartments and were most similar to the cecum. Similarity between the same compartments of different horses was low, further supporting that portions of the equine intestinal microbiota are unique to each animal.

## Abbreviations

PCR: Polymerase chain reaction; T-RF: Terminally labeled restriction fragment; TRFLP: Terminal restriction fragment length polymorphism

## Competing interests

The authors declare that they have no competing interests.

## Authors’ contribution

AS was involved in study design, carried out sample collection, laboratory work and drafted the manuscript. JW conceived the study, took part in the study design and helped with laboratory analysis. LA participated in study design and interpretation of data. HS participated in study design and coordination, and helped revise the manuscript. All authors read and approved the final manuscript.
